# BioCreative-IV virtual issue

**DOI:** 10.1093/database/bau039

**Published:** 2014-05-22

**Authors:** Cecilia N. Arighi, Cathy H. Wu, Kevin B. Cohen, Lynette Hirschman, Martin Krallinger, Alfonso Valencia, Zhiyong Lu, John W. Wilbur, Thomas C. Wiegers

**Affiliations:** Center for Bioinformatics and Computational Biology, University of Delaware, Newark, DE, USA.; Center for Computational Pharmacology, University of Colorado Denver School of Medicine, Aurora, CO, USA; The MITRE Corporation, Bedford, MA, USA; Structural and Computational Biology Group, Spanish National Cancer Research Centre, Madrid, Spain; National Center for Biotechnology Information, National Library of Medicine, Bethesda, MD, USA; Department of Biological Sciences, North Carolina State University, Raleigh, NC, USA

*BioCreative: Critical Assessment of Information Extraction in Biology* is an international community-wide effort for evaluating text mining (TM) and information extraction systems applied to the biological domain (http://www.biocreative.org/).The Challenge Evaluations and the accompanying BioCreative Workshops bring together the TM and biology communities to drive the development of practically relevant TM systems. One of the main goals of this initiative is that the resulting systems facilitate a more efficient literature information access to biologists in general, but also provide tools that can be directly integrated into the biocuration workflow and the knowledge discovery process carried out by databases. Beyond addressing the current barriers faced by TM technologies applied to biological literature, BioCreative has further been conducting user requirement analyses, user-based evaluations and fostering standards development for TM tool reuse and integration. This *DATABASE* virtual issue captures the major results from the Fourth BioCreative Challenge Evaluation Workshop, and is the sixth special issue devoted to BioCreative. Built on the success of the previous Challenge Evaluations and Workshops (BioCreative I, II, II.5, III, 2012) (1–5), the BioCreative IV Workshop was held in Bethesda, MD, on October 7–9, 2013.

BioCreative is distinct from other challenges in the bioNLP domain in how it selects its specific tasks, or tracks. From its inception, the organizers have worked with biocuration teams to define and evaluate tasks of importance to curation of the biomedical literature. Over the years, BioCreative has collaborated with curators from a variety of databases, including Gene Ontology Annotation (6), IntAct (7), MINT (8), BioGRID (9), Flybase (10), Mouse Genome Database (11), TAIR (12), CTD (13) and WormBase (14). This has enabled BioCreative to leverage existing standards, resources (especially, the knowledge captured in curated databases) and the expertise of the curators and to propose tracks that respond to their needs. As one example, in the BioCreative Workshop 2012, we reviewed descriptions of curation workflows from expert curated databases to identify commonalities and differences among these (15). One common theme was the need of semi- or fully-automated Gene Ontology (GO) curation techniques to assist database curators to rapidly identify relevant articles for GO curation, and as a result, a track for this topic has been included in the present challenge (see Track 4 below).

Challenge Evaluation tasks over the years have included ranking of relevant documents (‘document triage’), extraction of genes and proteins (‘gene mention’) and their linkage to database identifiers (‘gene normalization’), as well as extraction of functional annotation in standard ontologies [e.g. GO (16)] and extraction of entity relations [e.g. protein–protein interaction (17)]. Some TM tasks (e.g. gene normalization) are of fundamental importance to different applications, thus have been the subjects of multiple Challenge Evaluations to improve system performance. New tasks are also introduced to address new applications, tackling new entities, relationships, technical infrastructures and functional attributes (e.g. drug and disease). In BioCreative IV, we reintroduced the GO annotation task (see Track 4) similarly to the first BioCreative (18), but providing a comprehensively annotated corpus. We also introduced new tracks on interoperability of TM systems (Tracks 1 and 3), web service-based named entity recognition (NER, Track 3) and chemical/drug entity name recognition (Tracks 2 and 3).

In addition to the classic shared task model, which tends to focus on batch processing of large bodies of data and provides a valuable analysis of tool performance on component tasks of the biocuration workflow, starting with BioCreative III, BioCreative has created a track (Interactive Track) to explore interactions between biocurators and TM interfaces, as part of an investigation of utility, usability and use case generation for TM tools. Since then, the selection of tasks has been guided in part by the User Advisory Group (UAG, http://www.biocreative.org/events/biocreative-iv/CFP/#committee), made up of both curators from academia and consumers of curated information from biotechnology companies and pharma.

The general setting of the BioCreative Challenges and Workshops includes (i) the definition of user-centric relevant tasks; (ii) the preparation of data and infrastructure to evaluate the task by collaborating with domain experts and databases; (iii) the release of training data; (iv) the release of test data; (v) systems evaluation; and (vi) workshop and discussion of results and/or demo of the systems to provide feedback for improvements. Note that (iii) and (iv) only apply for shared tasks, whereas demos in (vi) apply mainly to the interactive task.

The BioCreative IV consisted of five Tracks:

- Track 1, BioC—The BioCreative Interoperability Initiative;
- Track 2, Chemical and Drug Named Entity Recognition (CHEMDNER)—Detection of mentions of chemical compounds and drugs;
- Track 3, Comparative Toxicogenomics Database (CTD)—Curation-related Interoperability and introduction of the concept of web services-based NER to identify gene/protein, chemical/drug, disease and action term mentions, supporting CTD curation in PubMed abstracts;
- Track 4, Gene Ontology (GO) curation—Development of automatic methods to aid GO curators in identifying articles with curatable GO information (triage) and extracting gene function terms and the associated evidence sentences in full-length articles;
- Track 5, Interactive Curation (IAT)—Demonstration and evaluation of web-based systems addressing user-defined tasks, evaluated by curators on performance and usability.

In addition, we organized a Metagenomics session to explore the TM needs of the metagenomics community. Metagenomics is the study of genetic material recovered directly from environmental samples; this new field is already having significant impact in other fields ranging from biodiversity to study of the human microbiome (microbial communities living on and in humans). Because metagenomics permits the study of naturally occurring microbial communities, accurate capture of metadata is critical to compare microbial populations across different conditions and environments (e.g. marine versus fresh water communities). However, this information is often embedded as free text descriptions in the literature or in text snippets in biological databases. The session consisted of a keynote followed by a panel of metagenomics researchers discussing their needs for capture of computable metadata and possibilities for a future BioCreative task related to metagenomics.

In total, BioCreative IV attracted 44 teams who participated and completed one or more of the five Tracks. Twenty-four unique teams were selected by the Organizing Committee to participate at the workshop, yielding five contributions in Track 1, nine in Track 2, four in Track 3, four in Track 4 and nine in Track 5. Nearly 70 participants attended the workshop, although around 20 more were expected who could not attend because of the US government shutdown. Also attending were nine members of the UAG, with representatives from many biocuration groups, particularly model organism databases, and from the pharmaceutical industry.

The Track 1 BioCreative Interoperability Initiative**—**Many researchers are building natural language processing (NLP) and TM tools. Yet these efforts tend to be singular, isolated and difficult to combine into larger, more powerful and more capable systems. The BioC format has been proposed as a simple extensible mark-up language format to share text documents and annotations. The proposed annotation approach allows a large number of different annotations to be represented including sentences, tokens, parts of speech, named entities such as genes or diseases and relationships between named entities (19). The core concepts are simplicity, interoperability and broad use and reuse of TM modules. This means there should be little investment required for learning to use a format or a software module to process that format. For the BioCreative IV Workshop, we invited teams to participate in the BioC initiative and contribute to this effort by preparing a BioC-compliant software module that could be seamlessly coupled with the rest of the BioC code and definitions, and that would perform an important NLP or BioNLP task. Teams were also expected to prepare a corpus or otherwise make data available, in the BioC format, to demonstrate and validate the function of their contribution. Eight different teams participated in the interoperability track at the BioCreative IV Workshop and contributed four new implementations of the BioC platform covering three new languages, 19 BioC compliant software tools, >25 annotated corpora converted to the BioC format and three web-based services accepting and producing their results in the BioC format. In addition, BioC was the medium used in the GO and CTD tracks and was also used by several teams for the IAT track. More details regarding these results may be found in the BioC Interoperability Track Overview paper and in papers by participating teams.

The Track 2 Chemical and Drug Named Entity Recognition (CHEMDNER)—There is an increasing interest to facilitate better access to information on chemical compounds and drugs (chemical entities) described in scientific articles. To achieve this goal, a crucial aspect is the automatic identification of mentions of chemical compounds in text. Recognizing chemical entities is crucial for subsequent text-processing strategies, such as detection of drug–protein interactions or the extraction of metabolic reactions. To improve the extraction of chemical entities from articles, the CHEMDNER task covered indexing documents with compounds (CDI subtask) and recognizing mentions of chemicals/drugs (CEM subtask). From the 65 registered groups, a total of 27 teams submitted results for the CHEMDNER task, 26 of them for the CEM subtask and 23 for the CDI subtask. Teams were provided with the manual annotations of 7000 abstracts to implement and train their systems and had to return predictions for the 3000 test set abstracts. When comparing exact matches of the automated results against the manually labeled Gold Standard annotations, the best teams reached an F-score of 87.39% in the CEM task and of 88.20% in the CDI task. This can be regarded as a competitive result when compared with the expected upper boundary, the agreement between two human annotators, at 91%. Owing to the relevance for the chemistry community, the overview and results for this task will be presented in a separate publication published in the Journal of Chemoinformatics.

The Track 3 Comparative Toxicogenomics Database (CTD) Curation-related Interoperability task—CTD (13) is a publicly available, manually curated resource that seeks to promote understanding of the mechanisms by which drugs and environmental chemicals influence biological processes and human health. To address the real need for NER tool interoperability and integration complexity abstraction, CTD invited TM teams to develop Representational State Transfer-based/BioC-compliant web services. These services would enable CTD to send text passages to their remote sites to identify gene/protein, chemical/drug, disease and action term mentions, each within the context of CTD's controlled vocabulary structure. Twelve teams submitted 44 web services with positive results, including top balanced F-scores for gene/protein, chemical/drug and disease NER of 61, 74 and 51%, respectively. Response times ranged from fractions of a second to >60 s per article. The results of Track 3 underscore the extraordinary ability of web services to insulate developers from the complexity of underlying computational systems, freeing them to focus on functional performance, and greatly simplifying TM pipeline implementation.

The Track 4 Gene Ontology (GO) Curation task—Although automatically predicting GO terms from research articles is not a new problem in TM, few studies have proven to be useful with regard to assisting real-world GO curation. The lack of access to full text, gold-standard training data such as evidence sentences, and few opportunities for interaction with actual GO curators has limited the advances in algorithm development and corresponding use in practical circumstances. The BioCreative IV GO task aimed at promoting and evaluating tool development for automatic GO annotation through literature mining. In collaboration with five model organism database groups [WormBase, FlyBase, RGD (20), MaizeGDB (21) and TAIR], the organizers provided teams with ∼4000 GO annotation-relevant text passages in 200 full-text papers; the establishment of such a textual evidence corpus has long been recognized as critical for TM algorithm development, but was never made available because of the high cost of curation. Seven teams participated in the GO task. From the team results, we find an overall improvement in performance in recognizing GO terms compared with the results of similar tasks in the past. Post-challenge analysis suggests future research directions of integrating domain knowledge for performance improvement and testing practical benefits of integrating TM tools into real-world GO annotation pipelines.

The Track 5 Interactive Curation (IAT) task—Featured demonstration and evaluation of interactive TM systems by biocurators. A user study was conducted by selected expert biocurators before the workshop that included time-to-completion on curation tasks and post-study surveys. In addition, a separate review of each system was performed by designing predefined tasks to get the first impression and assess specific functionalities via a user survey. Nine teams and >50 curators participated on this activity, with positive outcome and feedback from the biocuration community. Some of the systems increased curation efficiency based on the time spent in TM-unassisted versus TM-assisted curation. However, the actual curation time did not always reflect the user overall system satisfaction. For example, some curators noted that the system provided a nice interface with functionalities that, in the long run, would make the curation work more enjoyable. It was exciting to see the application of TM modules developed in previous BioCreative challenges. The task also promoted the generation of the annotated corpora in the BioC format, which is publicly available in the BioC Web site for other groups to use and further improve and refine this format. The interactive task has been an active generator of new interactions between TM systems and real users, which in some cases may lead to successful system adoption in biocuration.

The session on Metagenomics was organized to explore TM applied to a new area, namely metagenomics. The session included an invited talk from Dr. Pafilis (Hellenic Centre for Marine Research, Greece) on ‘SPECIES and ENVIRONMENTS: taxonomic name and environment descriptive term identification in text’, followed by a panel discussion on applications of TM to metagenomics with panelists from the metagenomics research community. The panelists included Prof. James Cole, Michigan State University; Prof. George Garrity, Names for Life and Michigan State University; Dr. Nikos Kyrpides, Joint Genome Institute; Dr. Folker Meyer, Argonne National Laboratory; and Prof. Lynn Schriml, University of Maryland School of Medicine; a sixth panelist, Dr. Tatiana Tatusova, NCBI, was not able to attend because of the government shut down. The panelists discussed use cases from their current research that highlighted the urgent need to capture computable metadata for metagenomics experiments. Types of metadata included taxonomic nomenclature (particularly challenging for microbial species), but also metadata associated with sample isolation source, organism habitat and organism phenotype. The metadata for metagenomics experiments can be found in the literature (in unstructured form), but also in semi-structured form in various databases, such as NCBI’s BioSample database. The ensuing discussion explored possible applications of TM—and sources of text—that might be suitable for a future BioCreative track focused on the needs of metagenomics research community.

The developments and contributions that BioCreative has stimulated since its inception are multifold including—

- **Advancing TM**: Scientifically, the workshops have had the effect of not only advancing progress on a variety of tasks, but have actually stimulated formulation of new capabilities in biomedical TM, such as gene/protein normalization (22, 23) and methods for Protein–Protein Interaction article classification and detection (24, 25). In BioCreative IV, chemical/drug, gene/protein and disease entity recognition have been studied with promising results, and also the new BioC format was introduced to promote interoperability.
- **Promoting interoperability and resource sharing:** New resources are produced to support the BioCreative shared tasks; these are publicly available in the BioCreative Web site with prior registration. The resources continue to be used by researchers for years after the original meeting for which they were created (26–30). In this regard, BioCreative IV has produced a collection of 200 full length papers with GO annotations with corresponding textual support, as well >1100 abstracts with gene/protein, chemical/drug and disease annotations, all in BioC format. In addition, there are deployable open access software systems that have been created to address the various BioCreative shared and interactive tasks, including 44 gene/protein, chemical/drug and disease NER web services introduced in BioCreative IV, all of which use BioC for interprocess communications, and many of which are expected to remain freely available. More importantly, we observed that some of the systems participating in the IAT task integrated tools that participated in previous BioCreative challenges, e.g. the PIE search (31, 32), GenNorm (33), GeneTUKit (34) and Ontogene (35, 36).
- Reaching new user communities: New communities have been approached to survey and respond to the TM needs in other domains. The CHEMDNER task was introduced in close collaboration with user groups with need on chemical-related information in biology. The Metagenomics panel helped to identify the urgent need to capture computable ecology- and biodiversity-related information for metagenomics experiments.
- **Bridging TM and user communities:** New collaborations between biocuration and TM groups are fostered in these workshops that could lead to adoption of the tools by the users.
- **Broadening research impact:** Finally, in terms of dissemination, there have been a large number of publications in peer-reviewed journals, including those published by participants in the meeting and those published by researchers who have continued working on the problems defined and data sets provided by the BioCreative organization after the meeting.

This *DATABASE* virtual issue includes overview papers describing Tracks 1, 3, 4 and 5 in BioCreative IV, as well as an overview of the Metagenomics panel session, and papers describing selected participating systems demonstrating significant contributions to biocuration. Note that results from Track 2 will be described in a separate publication given the large number of participating teams. The TM systems were selected based on performance, scientific advancements, innovation and significant impact, including their utility and usability as evaluated by biocurators.
